# New Approaches in the Study of the Pathogenesis of Urethral Pain Syndrome

**DOI:** 10.3390/diagnostics10110860

**Published:** 2020-10-22

**Authors:** Olga Streltsova, Anton Kuyarov, Muhhamad Shuaib Abdul Malik Molvi, Svetlana Zubova, Valery Lazukin, Ekaterina Tararova, Elena Kiseleva

**Affiliations:** 1 E.V. Shakhov Department of Urology, Privolzhsky Research Medical University, 10/1 Minin and Pozharsky Sq., 603950 Nizhny Novgorod, Russia; strelzova_uro@mail.ru (O.S.); kuyarov.anton@mail.ru (A.K.); msmolvi@mail.ru (M.S.A.M.M.); 2 N.A. Semashko Nizhny Novgorod Regional Clinical Hospital, 190 Rodionova St., 603126 Nizhny Novgorod, Russia; zubova.svetlana.65@yandex.ru; 3 Department of Medical Physics and Informatics, Privolzhsky Research Medical University, 10/1 Minin and Pozharsky Sq., 603950 Nizhny Novgorod, Russia; valery-laz@yandex.ru; 4 Nizhny Novgorod Regional Oncology Dispensary, 190 Rodionova St., 603126 Nizhny Novgorod, Russia; tararova-ea@mail.ru; 5 Institute of Experimental Oncology and Biomedical Technologies, Privolzhsky Research Medical University, 10/1 Minin and Pozharsky Sq., 603950 Nizhny Novgorod, Russia

**Keywords:** cross-polarization optical coherence tomography (CP OCT), ultrasound, urethral pain syndrome, epithelial atrophy, epithelial hyperplasia, inflammation, fibrosis, image evaluation

## Abstract

Introduction: Urethral pain syndrome (UPS) is still a pathology in which the diagnosis is formulated as a “diagnosis of exclusion”. The exact pathogenetic mechanisms are not yet fully understood and clear recommendations for the prevention and treatment of UPS are absent. Methods and Participants: A clinical and laboratory evaluation of 55 patients with established UPS included history taking, basic laboratory tests (e.g., complete blood count and clinical urine test), physical examination, uroflowmetry, and cystourethroscopy. Additionally, transvaginal ultrasound (TVUS) with compression elastography and cross-polarization optical tomography (CP OCT) were performed in 24 and 33 patients with UPS, respectively. The control group consisted of 14 patients with no complaints from the urinary system. Results: TVUS showed an expansion in the diameter of the internal lumen of the urethra, especially in the proximal region compared with the norm. Compression elastography revealed areas with increased stiffness (presence of fibrosis) in urethral and surrounding tissues. The performed CP OCT study showed that in UPS, the structure of the tissues in most cases was changed: trophic alterations in the epithelium (hypertrophy or atrophy) and fibrosis of underlying connective tissue were observed. The proximal fragment of the urethra with UPS underwent changes identical to those of the bladder neck. Conclusion: This paper showed that the introduction of new technology—CP OCT—in conjunction with TVUS will allow verification of structural changes in tissues of the lower urinary tract at the level of their architectonics and will help doctors understand better the basics of the UPS pathogenesis.

## 1. Introduction

The most common reason for women to seek medical attention is dysuria, and it is believed that in 40% of cases urethritis and/or urethral syndrome are involved [1]. According to the US National Institutes of Health, one third of women with chronic pelvic pain (CPP) have urethral pain syndrome (UPS) [2,3]. The European Association of Urology defines UPS as the occurrence of chronic or recurrent episodic pain lasting for more than 6 months, and felt in the urethra, in the absence of proven infection or other obvious local pathology. It is often associated with negative cognitive, behavioral, sexual or emotional consequences [4], as well as with symptoms suggestive of lower urinary tract, sexual, intestinal, or gynecological dysfunction [5].

The problem of pain in the urethra with unchanged urinalysis, the absence of any other clinical manifestations, and the absence of somatically explainable causes, is complex and ultimately remains unresolved, since the exact pathogenetic mechanisms are not yet fully understood [6,7,8,9]. Neither are there any clear recommendations for the prevention and treatment of UPS, as a result of which the only effective form of medical care today is symptomatic therapy—involving the continuing intake of strong pain medications, antidepressants, and anticonvulsants [4,9]. In the methodological recommendations on CPP, published under the auspices of the the Moscow Department of Health (dated 14 July 2016), it is noted that there is no specific accepted treatment for UPS [10]. The approach should be interdisciplinary and the treatment should be multimodal, with the general principles of chronic pain syndrome management being applied [11,12,13].

The close embryological relationship between the urethra and the bladder makes it likely that there are causes similar to ones connected with the development of painful bladder syndrome [14]. According to the classification of the International Association for the Study of Pain (IASP, 2019) the mechanism of CPP and possible causes of its occurrence may include vascular lesions, persistent inflammatory processes, or violation of the innervation of organs due to mechanical compression in the pelvic region, but often the reason is not clear [15].

The connective tissue matrix of organs plays a key role in the occurrence and persistence of pain, as shown by the number of studies [16,17]. It is believed that connective tissue, as well as performing its supporting, protective and trophic functions, acts as a network-wide mechanosensitive signaling system—as a global unifying network [16,18].

Thus, it can be surmised that the above reasons for the development of CPP could be associated with factors that affect the state of the connective tissue matrix of the lower urinary tract. However, there are currently no methods for adequate, appropriate study of the structure of urethral tissues. According to the standards for examination of patients with CPP when using the UPOINT (Urinary, Psychosocial, Organ Specific, Infection, Neurologic/Systemic, Tenderness of Skeletal Muscles) classification [19] in the urology domain, the recommended list of examinations includes keeping a urination diary, cystoscopy, and the use of ultrasound (US) and uroflowmetry, while for complaints involving the urethra, urethroscopy is recommended. These methods allow only indirect assessment of the urethral tissues. Objective evaluation and accurate diagnosis of a disease that does not cause any visual changes, and results from a “diagnosis of exclusion” when using standard instrumental diagnostic methods, is important for understanding the pathogenetic aspects of the disease. In this work, we used traditional diagnostic methods, including US and uroflowmetry, and the non-traditional methods of ultrasound elastography (USE) and cross-polarization optical tomography (CP OCT) to study changes in the functioning of organs and their structure in UPS in comparison with the norm, and assessed the role of background diseases in the development of UPS.

The USE is a medical imaging modality that measures tissue mechanical properties by monitoring the response of tissue to acoustic energy [20,21]. In clinical settings, USE is emerging as a powerful tool for imaging and quantitatively monitoring cancer and fibrosis [22]. It provides a rapid visualization of the tissue elasticity using color-coding mode, even in organs deep within the body. Recently, USE is applied especially on the breast and liver, but the technique has been increasingly used for other tissues including the thyroid, prostate, lymph nodes, gastrointestinal tract, kidney, spleen, pancreas, and the musculoskeletal and vascular systems [22,23]. There are several USE techniques used in clinical practice, but USE with strain (compression) being the most common one allowing real-time visualization of the elastographic map on the screen [24]. With regard to the study of the urethra in UPS, the method can be useful for detecting fibrous changes in the urethral wall and adjacent tissues.

In general, OCT is similar to the ultrasonic technique, except for using light instead of sound and is centered on interferometry in the near-infrared range of wavelength (700–1300 nm) [25,26]. It measures the time delay and amplitude of backscattered light. The aim of the OCT technology is to perform a real-time, in vivo, optic biopsy, with direct label-free visualization of the histological structure of the human tissues at the level of the general architectonics to a depth of 1.5 mm [27]. High spatial resolution (5–15 µm) and performance simplicity with minimal expertise are the main advantages of OCT in contrast to US. The endoscopic nature of OCT probes not only enhances patient comfort and safety but also makes it especially suitable for assessing narrow tubular organs as well as for using standard guidewires for examining deeply located objects in the body [28].

CP OCT is a functional extension of OCT that enables the detection of changes in the state of polarization of light caused by birefringence and coupling between two polarization states due to scattering in the random media (cross-scattering) [29]. As a result, two types of images are obtained simultaneously: in the initial (co-) polarization and orthogonal (cross-) polarization, which allow assessing isotropic (cells) and anisotropic (collagen and elastic fibers of connective tissue) structures separately [30,31]. This is important in cases when precise observation of only connective tissue structures is needed.

The goal of the study was to assess the condition of the tissue in the female urethra in UPS, by using non-traditional methods for this pathology—compression US and CP OCT.

## 2. Materials and Methods

### 2.1. Patients

In total, 69 female patients were enrolled in this study: 55 with established UPS (“UPS” group, aged from 21 to 66 years) and 14 with a healthy urethra as a control group (“Norm” group, aged from 24 to 62 years). Patients with UPS received treatment in the urology department of the N.A. Semashko Nizhny Novgorod Regional Clinical Hospital between 2014 and 2019.

Inclusion criteria for the UPS group consisted of: age 18 years and older; presence of recurrent episodic pain localized in the urethra lasting more than 6 months; absence of infectious lesion or obvious organ pathology [32]. Exclusion criteria for UPS group were: age under 18; the presence of inflammatory processes in the lower urinary tract; the presence of tumors of the pelvic organs; radiation damage to the pelvic organs; pregnancy; lactation. The control group included women whose age was 18 years and older, with no detected pathology and complaints from the lower urinary tract. Otherwise, people were excluded from the study.

This study was approved by the review board of the Privolzhsky Research Medical University (Protocol #6 from 28 April 2020). The research was carried out within the framework of the RFBR project #19-07-00395, agreements 1236/19 from 11 April 2019 and #1365/20 from 30 March 2020. Informed consent to participate in the study was obtained from the participants. All conducted studies and the number of patients included are presented in Table 1.

### 2.2. Transvaginal US

Transvaginal US (TVUS) was performed using a Philips Epiq5 system (Philips Ultrasound., Inc., 22100 Bothell-Everett Highway, Bothell, Washington, 98021-8431, USA). The sensor was inserted directly into the vagina, allowing visualization of the state of the bladder neck and urethra (assessment of their structure, the condition of their walls, and the width of the internal lumen) and detecting abnormalities in the structure of the urethra compared with the norm. This was also the first study in which patients with UPS underwent compression elastography of the adjacent urethral tissues. Compression elastography is a technique that displays the relative deformation of tissues in the form of their color mapping in real time [33]. When the tissue is subjected to an external force (deformation), the harder/denser areas of the tissue exhibit relatively less compression than the softer areas [24]. In our study, on the USE images, the adjustment scale was set to display the harder areas in blue, with the softer areas appearing in red [34].

### 2.3. CP OCT Study and Image Analysis

**Figure 1:**
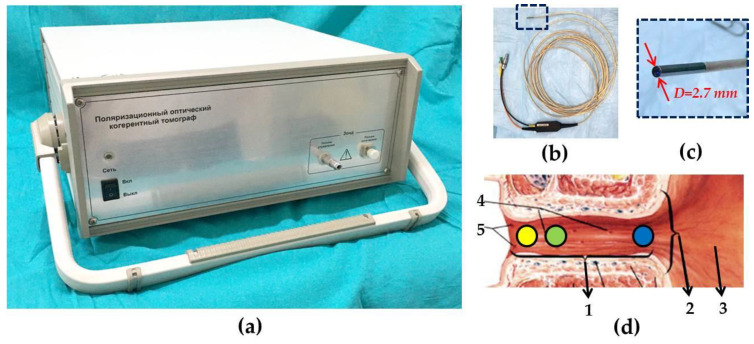
Cross-polarization optical tomography (CP OCT) device and areas under study shown on a diagram of the female urethra. (**a**) CP OCT device; (**b**) Flexible endoscopic forward-looking CP OCT probe; (**c**) Enlarged tip of the probe from (**b**). (**d**) Drawing of the urethra where it transitions to the bladder. Here, the circles indicate the locations from which CP OCT images were obtained in the proximal (blue), middle (green), and distal parts of the urethra (yellow) [37]. 1—urethra, 2—neck of urinary bladder, 3—triangle of urinary bladder, 4—lacunae and openings of urethral ducts, 5—openings of paraurethral Skene’s ducts.

Time-domain device “Polarization-sensitive optical coherence tomograph OCT-1300U” (BioMedTech LLC, Nizhny Novgorod, Russia) (Figure 1a), that provides two image acquisition in co- and cross-polarizations was used in the study [29,35]. The device is approved for clinical use (product license №FCP 2012/13479 of 30 May 2012) and is equipped with replaceable endoscopic probe (Figure 1b,c). It has the following characteristics: the radiation source is a superluminescent diode, of operating wavelength 1310 nm, spectrum width 100 nm, axial resolution 15 μm, lateral resolution 25 μm, and radiation power at the object 3 mW. OCT image size in each polarization is 1.8 × 1.3 mm (width × height), image acquisition time is 2 s. Due to the presence of a flexible endoscopic probe with an outer diameter of 2.7 mm, the examination of the urethral tissue could be carried out simultaneously with cystoscopy through a standard endoscope. Our group’s application of the CP OCT method to the study of the female urethral wall in patients with UPS, is a global “first” [36].

From 4 to 13 images were obtained from each patient: of the bladder neck and three regions of the urethra (Figure 1d) at the 6 o′clock position corresponding to a conventional clockface, and, if possible, with other additional images of the urethra in the three directions (9, 12, and 3 h of the clockface).

In the “UPS”/”N” groups, 169/58 CP OCT images were obtained, which included 43/16 CP OCT images of the bladder neck, as the section closest to the urethra and therefore potentially involved in processes occurring in the proximal urethra and 126/42 CP OCT images of the urethra (its proximal 41/14, middle 40/12, and distal 45/16 regions) (Table 2).

A visual assessment of the CP OCT images of the bladder neck and urethra was performed by two readers. The objects of interest were the epithelium and the state of the connective tissue structures of the urethra in patients with UPS, relative to the normal state of these structures. In the epithelium, the thickness was assessed as: normal, thickening (hyperplasia), or thinning (atrophy); in the connective tissue stroma, attention was paid to the presence of any element in the images corresponding to an inflammatory process or fibrosis. The CP OCT features of inflammation were: (1) lack of clarity of the border between the first (epithelial) and the second (connective tissue) layers, (2) the absence of horizontal ordering of the structures that are representative of the norm, and (3) the presence of any indistinctness in their images, which would correspond to cellular tissue infiltration. Significant thickening of the connective tissue layer, up to the lower border of the image with maintaining a high signal level was considered a sign of fibrosis [36,38]. Before qualitative evaluation of CP OCT images readers were trained by training test. After an independent blind visual assessment of the CP OCT images, the «UPS» group was divided into 2 age subgroups: patients under 50 and those over 50.

### 2.4. Statistical Analysis

The statistical analysis was performed using IBM SPSS Statistics software, V20 (IBM Corporation, Somers, NY, USA). The inter-reader reliability was calculated using the Fleiss’ kappa (κ) coefficient: κ > 0.8—perfect agreement; 0.7 ≤ κ < 0.8—substantial agreement; κ < 0.7—poor agreement.

## 3. Results

### 3.1. The Role of Background Diseases in the Development of UPS

An analysis of concomitant pathology in patients with UPS, identified by their history is presented in Table 3. From Table 3 it follows that the predominant area of comorbidity was gynecological (70.9%). Hormonal abnormalities (94.8%) were found in 24 sexually active women in the pre-menopausal period, as well as in 13 women of the menopausal period; inflammatory diseases of the female genital area of bacterial and viral etiology were also present (76.9%).

Anamnesis of upper respiratory tract pathology, more common in adolescence, was recorded in 67.2% of women, of whom the bulk of patients (64.9%) reported frequent viral diseases or herpes infection. The premorbid background in patients with UPS was neurological pathology (63.6%), and these are diseases associated with the involvement of the peripheral nervous system and as is important, with the state of the psycho-emotional sphere.

Each patient suffering from UPS had 2.94 (162/55) cases of comorbidity. Thus, the role of other factors in the presence of foci of chronic infection in the body, and a decrease in immune defense factors, as a comorbid background for the development of UPS, cannot be denied, since the presence in the patients’ history of inflammatory diseases of the respiratory tract, gastrointestinal tract, urological and gynecological organs was revealed.

### 3.2. Results of Cystoscopic Examination

In 32.7% of cases (18 out of 55), clinical manifestations of UPS were combined with urinary pain syndrome. Low-volume (less than 300 mL) urination was reordered. The number of urinations exceeded 12 per day. Pains over the womb were present. During cystoscopy in patients of the “UPS” group, the bladder mucosa was unchanged—shiny, pale pink, while, in 16 cases (29.0%), there was a slight hyperemia in the bladder neck. A picture corresponding to interstitial cystitis—the presence of glomerulations in the mucous membrane of the bladder after the hydrodistension procedure, was found in 23.6% (*n* = 13).

### 3.3. Uroflowmetry Results

In 72.7% of cases (40 out of 55), there was a decrease in the urination rate to 13.7 ± 3.2 mL/s in combination with low-volume urination while the normal values of the urination rate for women are 23–32 mL/s [39]. The average volume of excreted urine was 172 ± 33 mL.

### 3.4. Results of TVUS Research

**Figure 2:**
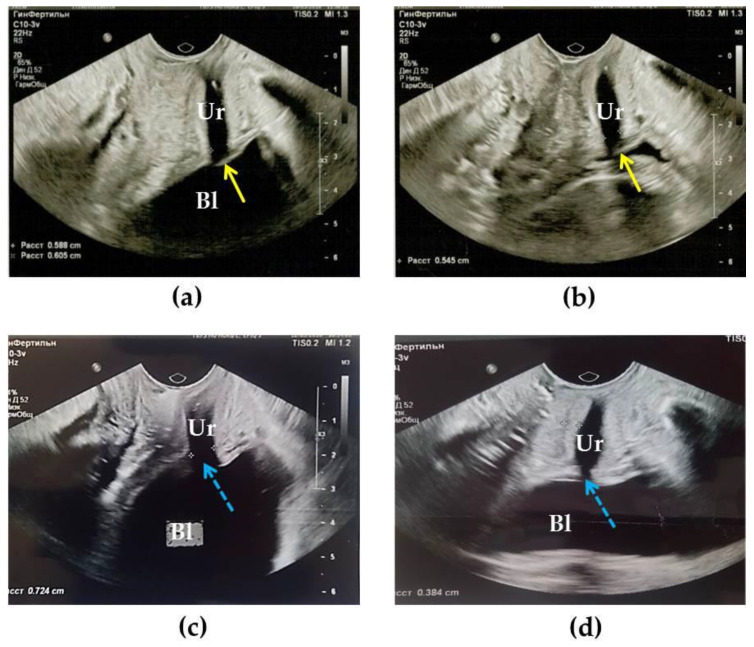
Transvaginal ultrasound (TVUS) of the urethra and adjacent tissues in normal conditions and with UPS. (**a**,**b**) A healthy woman 30 years of age before (**a**) and after (**b**) urination. The urethral tongue closes the opening to the urethra, as indicated by the yellow arrow; (**c**) Patient K., 30 years old, with a UPS disease duration of more than 10 years; (**d**) Patient Z., 38 years old, over 13 years of illness. In both cases, with UPS, the urethral tongue is indistinguishable and the gaping opening at the transition of the bladder into the urethra is indicated by the blue dashed arrows. Bl—bladder, Ur—urethra.

The results of TVUS studies showed that in the norm group in women, the urethra looks like a tube with a uniform lumen diameter without dilatations and contractions, which was 4.6 ± 0.6 mm, wall thickness 4.8 ± 1.1 mm. According to research by a group of authors [40], normally, the outer diameter of the urethra is 10.0 mm, the inner lumen of the urethra is closed during TVUS or 0.3 mm (Figure 2a,b). According to the authors [41], who conducted a study with an intraurethral sensor, the thickness of the urethra in the proximal section was normally 3.7 mm.

In women with UPS (*n* = 24), the structural features of the urethra were revealed: the urethra was funnel-shaped (Figure 2d), opening to the bladder. The internal lumen of the urethra in the proximal segment was expanded to 5.9 ± 2.1 mm. At the same time, 44% of patients had an expansion up to 7.5 ± 0.5 mm, in 56% up to 5.5 ± 0.5 mm. The thickness of the urethral walls in our study averaged 3.6 mm (from 2.4 to 6.0 mm). Thus, in all patients with UPS, an increase in the diameter of the internal lumen of the urethra, especially in the proximal region, was recorded.

In 7 (29.1%) cases, pathological changes were recorded in the urethral tongue, a cavernous structure that, as the bladder fills, normally increases in volume due to becoming engorged with blood and, together with the sphincter trigonalis, closes the exit from the bladder into the urethra. With the contraction of the urethra the posterior semicircle of the bladder neck is pressed against the anterior wall of the urethra and this closes its internal opening [42]. In patients with UPS, an absence of urethral tongue visualization (Figure 2c,d), or the absence of its adherence to the entrance to the urethra, was revealed. No residual urine was found in patients with UPS.

**Figure 3:**
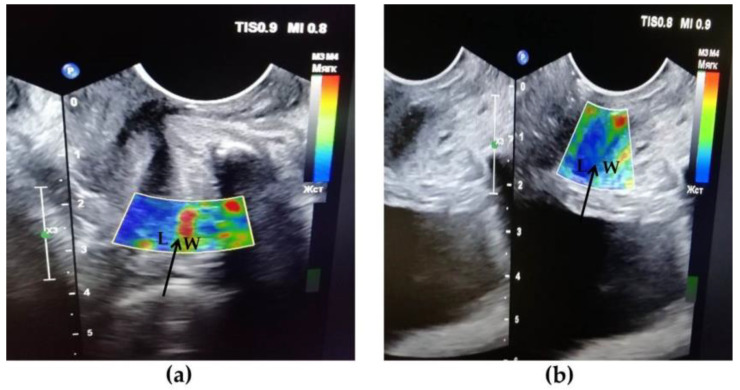
Compression ultrasound (US) in the normal condition (**a**) and in UPS (**b**). (**a**) Normally, the urethral wall is softer (red color) than in UPS (**b**) (predominance of green and blue colors). L—lumen of the urethra, W—urethral wall. The black arrow indicates the border between the lumen of the urethra and its wall.

Compression US of the urethra and adjacent tissues of patients with UPS in the proximal and middle regions showed a significant predominance of areas colored blue, indicating tissue stiffness and rigidity (Figure 3b) compared to the norm, where no blue color was observed (Figure 3a). Thus, our studies confirm the presence of fibrosis of the tissues surrounding the urethra in UPS.

### 3.5. Results of CP OCT Study

**Figure 4:**
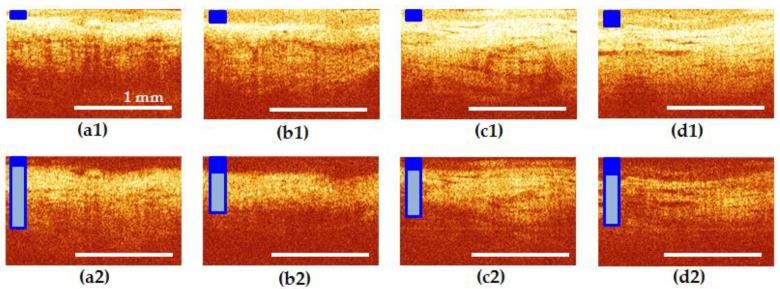
CP OCT images of the bladder neck (**a**) and three segments of normal urethra (**b**–**d**): (**b**) proximal; (**c**) middle; and (**d**) distal. The first row shows co-polarization images, the second row shows corresponding cross-polarization images. The first (epithelial) layer and its thickness are marked in all images with a dark blue rectangle. The second layer (connective tissue of lamina propria) in (**a2**–**d2**) is indicated by a vertical rectangle in light blue color: its height shows the average height of the layer and the blue color inside the frame indicates its normal condition.

CP OCT images of all sections of the female urethra in the norm are structural. In co-polarization images (Figure 4a1–d1), the epithelium is clearly visualized in all areas of interest, its border contrasting with the underlying mucous layer. The epithelial layer and its thickness are marked in Figure 4a–d with dark blue rectangle. The signal from the connective tissue in the cross-polarization images (Figure 4a2–d2) is of medium intensity, has a horizontal orientation; in the middle and distal segments of the urethra, single, gland-like lacunas with clear contours can be determined. In cross-polarization, the OCT signal is determined mainly by the collagen fibers of the connective tissue layer, therefore, only this layer of the urethral wall is clearly visible in such images, and the epithelium and muscles are not visualized.

In the normal group, there were no changes in the visible thickness in the zones of interest (Figure 4a1–d1). However, in women over 50 years old, a tendency of the epithelium to atrophy was revealed, which can be explained by the influence of hormonal changes. The connective tissue stroma generated approximately the same signal level in the cross-channel, without any extensive dark or bright areas and occupied 40–50% of the entire image height (Figure 4a2–d2), connective tissue is marked by vertical rectangle in light blue color: its height shows an average height of the layer and the light blue color inside the frame indicates its normal condition).

**Figure 5:**
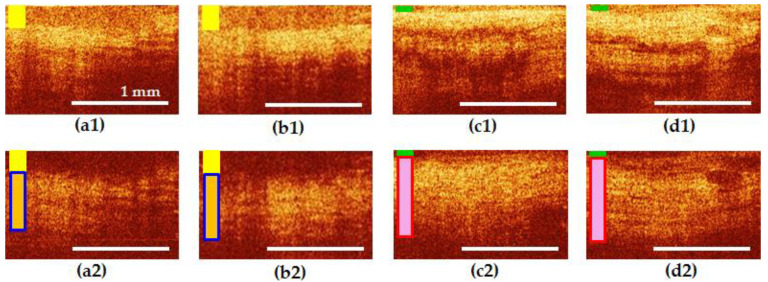
CP OCT images of the bladder neck (**a**) and three segments of the urethra (**b**–**d**) in patient I., 22 years old with UPS lasting 5 years. (**b**) Proximal; (**c**) middle; and (**d**) distal parts of the urethra. The first row shows co-polarization images, the second row shows corresponding cross-polarization images. (**a**,**b**) Epithelium hyperplasia is marked with yellow rectangle. Connective tissue in (**a2**,**b2**) have normal thickness (dark blue frame of vertical rectangle), but signs of inflammation (yellow color inside the rectangle); (**c**,**d**) Epithelial atrophy is marked with green rectangle. Connective tissue in (**c2**,**d2**) has increased thickness (red frame of vertical rectangle) and signs of fibrosis (pink color inside the rectangle).

**Figure 6:**
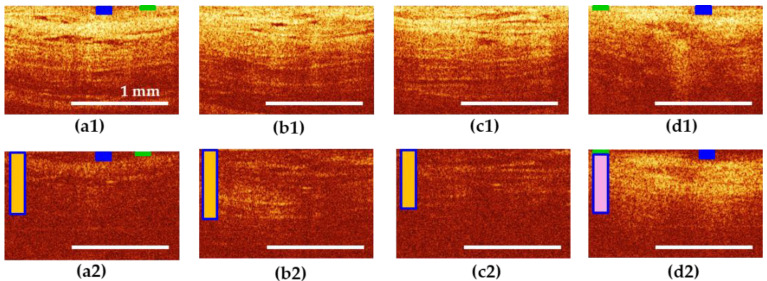
CP OCT images of the bladder neck (**a**) and three segments of the urethra (**b**–**d**) in patient E., 60 years old with UPS lasting 5 years. (**b**) proximal; (**c**) middle; and (**d**) distal parts of the urethra. The first row shows co-polarization images, the second row shows corresponding cross-polarization images. (**a**,**d**) Epithelium has signs of norm (blue rectangle), but mostly atrophic (green rectangle); (**b**,**c**) total epithelial atrophy is observed. (**a**–**c**) Connective tissue have normal thickness (dark blue frame of vertical rectangle), but signs of active inflammation (yellow color of vertical rectangle); (**d**) connective tissue has normal thickness (dark blue frame of vertical rectangle), but signs of fibrosis (pink color of vertical rectangle).

Visual analysis of CP OCT in the UPS group revealed that, in terms of the characteristics of the epithelium and connective tissue, the proximal part of the urethra was more similar to the bladder neck than to the middle and distal parts of itself. Examples are shown in Figure 5 and Figure 6.

Figure 5 shows an example of patient I., 22 years old with UPS lasting 5 years. Epithelial hyperplasia is visible in the bladder neck and the proximal urethra (Figure 5a1,b1), the yellow rectangle), the border of the epithelium with the underlying connective tissue layer is blurred, indicating the presence of inflammatory processes in these tissues (Figure 5a1,b1). The signal from the connective tissue structures in cross-polarization has a noticeable local decrease in intensity caused by the shadows of dilated blood vessels and by tissue edema (Figure 5a2,b2), the vertical rectangle in yellow color shows tissue inflammation, dark blue frame indicates normal thickness of the layer). In the middle and distal parts of the urethra, by contrast, thinning of the epithelium is noticeable (Figure 5c1,d1), green rectangle), while in the middle part, the border with the underlying connective tissue layer is clear (Figure 5c1)).

The connective tissue layer is thickened (Figure 5c2,d2), the red frame of vertical rectangle indicates increased thickness of the layer, pink color indicates signs of tissue fibrosis and looks more homogeneous in structure (Figure 5c2) than in the non-pathogenic case (Figure 4c2,d2). In this subgroup of patients, a thickening of the connective tissue layer in cross-polarization to occupy over 60% of the image height was observed in 44.4% (32 CP OCT images out of 72). In this case, an increase in the OCT signal was observed in all the images.

Figure 6 shows an example of patient E., 60 years old with UPS lasting 5 years. In the bladder neck and proximal urethra (Figure 6a1,b1), as well as in the rest of the urethra (Figure 6c1,d1), the epithelium is atrophic, and in places where it is partially preserved (Figure 6a,d, blue and green rectangle), the border of the epithelium with the underlying connective tissue layer is blurred (Figure 6a1,d1)). The signal from connective tissue structures in cross-polarization is weak, presumably due to severe tissue edema (Figure 6a2–c2), vertical rectangle in yellow color shows tissue inflammation, dark blue frame indicates normal thickness of the layer). In the distal urethra, on the other hand, the connective tissue layer exhibits cross-scattering, but appears homogeneous in structure (Figure 6d2), pink color of vertical rectangle indicates fibrosis) compared to normal (Figure 4d2). In this subgroup of patients, thickening of the connective tissue layer in cross-polarization to over 60% of the image height was observed in 46.7% (28 CP OCT images out of 60): 71.4% of them with an increase in the OCT signal (20 of 28), while 28.6% (8 out of 28) showed a weakening of the signal.

The inter-reader reliability in qualitative evaluation of CP OCT images was 0.93 that indicates high concordance between the two readers. Disagreements were observed in cases of focal epithelial atrophy (an example, Figure 6a,d): one of the readers rated the epithelium as atrophic, the other as normal. In other cases, the answers were completely the same.

The results of the incidence of the bladder neck + proximal conditions are presented in Table 4. 132 CP OCT images obtained at the ‘6 o′clock position’ from 33 patients were analyzed.

It was revealed that changes in the epithelium of the bladder neck and proximal urethra—hyperplasia or atrophy, which differed from the middle and distal segments of the urethra—coincided in 22 cases out of 33, representing 68.8%. Hyperplasia was identified in 34.4% of cases (*n* = 11) as well as atrophy in 34.4% of cases (*n* = 11). It is noteworthy that in women over 50 years of age (*n* = 15), changes in the analyzed area were more common—93.3%, compared with women of reproductive age (*n* = 18)—44.4%. It can be surmised that hormonal levels undoubtedly play a role in changing the state of the tissues of the bladder neck and urethra.

Of the 11 cases of hyperplasia detected in the proximal urethra, only in the case of the epithelium was there also thickening in the middle and distal urethra. In other situations, atrophy was recorded—our cases, while, in six the epithelium was of normal thickness. In the presence of atrophy in the proximal urethra (*n* = 11), atrophy was recorded in the underlying regions—five cases, while the epithelium was of normal thickness in six cases.

Thus, the CP OCT method allowed us non-invasively to determine the state of the epithelium and connective tissue structures of the bladder neck and urethra in vivo. It was shown that with UPS, the structure of the tissues in most cases is changed. In this case, the proximal fragment of the urethra with UPS undergoes changes identical to those of the bladder neck.

## 4. Discussion

UPS is still a pathology in which the diagnosis is formulated as a “diagnosis of exclusion”. It is not specific for women, as it can also occur in men [43], but in this case, it is customary to speak about prostatic, scrotal, or penile chronic pelvic pain [8]. A large number of studies have been devoted to the study of UPS and chronic pelvic pain in men [44,45,46,47,48], while UPS in women has been studied significantly less [3,6]. Therefore, one of the motivations was to conduct research on the female urethra. In any case, the problems of correct and quick diagnosis and the appointment of effective treatment for UPS in men there remain the same [5]. An idea to diagnose male UPS by using ultrasound and CP OCT devices seems to be reasonable and appropriate.

Despite significant global use of OCT in many fields of medicine [49,50,51,52,53], in urology, our study demonstrated the first use of this technique for examining the urethra [36]. This paper shows that the introduction of new technology—CP OCT—in conjunction with TVUS allows verification of tissue changes and assessment of the structures of the connective tissue matrix of the lower urinary tract at the level of their architectonics.

According to TVUS, in our study, women with UPS had an enlarged internal lumen of the urethra in the proximal segment—on average of 5.9 ± 2.1 mm. According to the literature, with an intraurethral ultrasound study performed on sectioned material, the inner diameter of the proximal segment of the urethra at distances of 10, 15, and 20 mm from the neck was 3.73, 4.18, and 2.64 mm, respectively [41]. In another study, when measuring the internal diameter of the urethra using TVUS in women with urinary incontinence [54], the diameter in the middle third of the urethra in the control (healthy) group of patients was 4.7 ± 1.1 mm. Thus, we have recorded an increase in the diameter of the internal lumen of the proximal urethral segment in all patients with UPS. Normally, upon initiation of urination, the mechanism for opening the funnel-shaped depression in the bladder neck is associated with contraction of the muscles of the deep triangle and of muscles located anterior to the internal opening of the urethra, as well as with the simultaneous contraction of the longitudinal muscle fibers of the urethra [42]. This means, we can assume the presence of insufficiency of these muscle groups in UPS.

Trophic disorders recorded by CP OCT in the epithelium of the urethral neck and the proximal segment of the urethra were more common in women over 50 years of age—in 93.3%, indicating their dependence on the patient′s hormonal background. The hormonal dependence of a number of urinary disorders is explained in [55,56]. In these works, it was shown that in the deep layers of the mucous membrane of the urethra there is a powerful venous plexus, and that this has a large number of anastomoses with the venous uterovaginal plexus. At the same time, the work of Petros et al. [57] indicated that the epithelium of the urinary system (urothelium) acts as a mechanoreceptor, using its sensitive nerve endings, and that it controls the activity of the afferent nerves, so this may contribute a pathogenetic component of chronic pelvic pain, and of urethral syndrome in particular.

Using the CP OCT method, we have previously shown that the thickness of the tissue of the urethral membrane in women is dependent on age [58]. The work reported that, with UPS, there are corresponding tendencies towards thinning of the epithelium and an increase in the thickness of the connective tissue matrix of the bladder neck, as occurs in women without pathology of the urological sphere, but that these processes proceed at a higher rate.

The recorded changes in the thickness of the epithelium are undoubtedly associated with the state of the connective tissue matrix of the subepithelium of the structural components. The compaction of the walls of the urethra and surrounding tissues that we have revealed using elastometry data, as well as in our earlier CP OCT data on the state of the connective tissue matrix of the urethra during UPS [36], indicate the presence of fibrosis processes both within the wall of the urethra and around it, the cause of which, at present, is not clear. Our studies have previously shown that the state of the urethral tissues in UPS is not normal, with changes in the urethral tissues occupying an intermediate place between the norm and the changes seen in chronic bacterial inflammatory processes [36].

Changes in the state of the connective tissue can lead to a decrease in the sensitivity of the stretch receptors at the base of the bladder, affecting the functionality results [57], in particular, influencing the uroflowmetry data that we obtained. The results of the uroflowmetry allow us to assume the presence of functional disorders of the urethra in women with UPS. Considering the indices of the normal values of the urination rate for women, which are 23–32 mL/s [40], our results of uroflowmetry showing 13.7 ± 3.2 mL/s are likely to be associated with anatomical changes that are not detected in standard clinical studies, or with dysfunctional and/or obstructive urination due to an overactive urethra. However, it is known that the presence of symptoms of urinary disorders is not a reliable marker of pathological processes [40]. We are continuing our research in this direction.

There is reason to believe that the cause of the development of chronic inflammatory processes in UPS is located in the tissues of the urethra and, accordingly, this serves as an additional stimulus for the occurrence of disorders of the microcirculation, innervation, and functioning of the urethra, indirectly influencing the appearance of pain. Our anamnestic data on the presence of a prevailing gynecological pathology of inflammatory genesis suggest that the cause of such changes in the tissues of the bladder and urethra may be viral-bacterial associations in the tissues of the organs of the gynecological sphere. This aspect requires more detailed study. At present, the effect of the translocation of microorganisms in the tissues of the urinary system, vagina, and intestines has been proven in cases of upper urinary tract infection [59], although research in this area is ongoing. Analyses of the composition of the microflora of urine and of the large intestine in cases of infection of the lower urinary tract have also indirectly confirmed the presence of a translocation mechanism in microorganisms [59].

It is known that the close anatomical connection of the bladder, urethra, and vagina provides associated functional mechanisms for the urination process. A component of this mechanism is illustrated by the fact that in the distal urethra the circular fibers of the striated sphincter are transformed into loop structures, the ends of which are woven into the framework of the anterior vaginal wall [55]. According to the anamnesis, hormonal disorders, inflammatory diseases, and surgical interventions on the pelvic organs, which could result in dysfunction of the muscles of the urethra and vagina, were found in 70.9% of patients with UPS who were interviewed. At the same time, it is known that functional disorders, on their own, can generate pain [60]. The results of our study indicated that a reason for the development of pain and chronic dysuria in patients with UPS may be failure of the structures of the internal urethral sphincter. This sphincter is formed by the muscles of the external muscular layer of the bladder that pass into the urethra in the bladder neck region, forming spiral structures, occupying about 20% of its length. In the present study on TVUS, 29.1% of women were found to have an insufficiency of structures, namely the urethral tongue, in the area of this sphincter. This fact requires further research.

Thus, it has been shown that there are many factors that cause persistent long-term pain in the urethral region, or that contribute to the intensification of pain, some of which have yet to be studied. Given the non-obviousness of the causes of UPS, new research protocols and additional imaging and diagnostic methods are required for a comprehensive examination of such patients, without focusing only on their pathologies in the urological field.

The main shortcoming of our study was its retrospective nature and moderate patient number in the norm group. Therefore, our results are not definitive and require confirmation on a larger number of patients. However, we identified certain new patterns in patients with UPS compared with healthy women. We can suggest including our approach—the combined study of patients with UPS by TVUS/compression US and CP OCT—in the daily practice of urologists in order to undergo validation and prospective–comparative clinical trials.

Another drawback of our study was the lack of histological verification of CP OCT data. We proceeded from the results of our previous study [36], where the morphology of the female urethra was analyzed on cadaveric material and compared with CP OCT images. This fact and numerous studies on CP OCT visualization of mucous membranes in health and pathology [30,31,52,61,62] afford ground for confidence in the interpretation of CP OCT features, such as changes in the epithelium thickness and the state of connective tissue.

As soon as the limitations of the used methods are concerned, it is necessary to compare their imaging depth and resolution. With a fairly low resolution of TVUS, the advantage of the method is a sufficient imaging depth. The technique allows observing the entire urethra, estimating its size and shape, identifying concomitant pathologies in adjacent organs, and conducting a functional study (compare the state of the urethra and bladder before and after miction). In addition to the USE mode, which is used in this paper, the method also makes it possible to study the blood supply to the pelvic organs, which is an important part in the pathogenesis of UPS.

One of the limitations of the CP OCT method is the small depth of tissue visualization, namely, the inability to fully assess the muscle layer located deeper than the connective tissue. On the other hand, tissue imaging to a depth of 1–1.5 mm is an advantage over urethroscopy, which allows the assessment of the urethral mucosa only from the surface. The forward-looking CP OCT probe used in this study allows visualizing the urethral wall in a certain place, while it would be optimal to study the urethral mucosa along its entire length, for example, when using rotary or needle OCT probes with manual scanning [28]. Despite this, the advantage of CP OCT is the rapid assessment of the urethral wall structure at the tissue level: the high resolution of the method (5–15 μm) is sufficient for the rapid assessment of epithelium and connective tissue—structures that play a key role in the emergence of UPS [63].

As a prospect, we intend to continue research on the pathogenesis of UPS by adding neurophysiological methods for diagnosing lesions of the pudendal nerve and sacral pathways, assessing the hormonal status of women, and studying the microflora of the tissues of the urethra and the bladder neck.

## 5. Conclusions

For the first time in the case of UPS, the layered structure of the urethral wall was investigated in vivo using CP OCT to assess some of the pathogenetic aspects of the development and progression of this disease. The CP OCT method covers the range of possibilities of traditional cystoscopy and allows information to be obtained about the state of the urethral tissues that cannot be adequately assessed during cystoscopic examination alone. The predominant changes in the tissues of the urethra are fibrosis of the subepithelial structures and trophic changes in the epithelial layer. In 68.8% of cases, the “behavior” of the tissues of the proximal segment of the urethra coincided with changes in the bladder neck. The importance of the in vivo acquisition and operative analysis possible with CP OCT in combination with TVUS/compression US data in patients with UPS is beyond doubt.

Deep objective analysis of tissues can reveal the basis of pathogenesis. Real-time visualization of structural changes in the tissues of the urethra (epithelium, connective tissue, muscle layer, vasculature, and paraurethral glands) is important because it influences the final diagnosis, understanding of the pathogenesis of the disease and treatment tactics. An analysis of the comorbidities of patients with UPS showed that inflammatory gynecological diseases can become a premorbid background/one of the triggering mechanisms for the development of UPS.

## Figures and Tables

**Table 1:** Clinical and laboratory methods for patient’s examination and number of included patients.

Type of Study	Purpose of the Study	Number of Patients in the UPS Group	Number of Patients in the Norm Group
1. History taking	identification of the presence of any previously transferred concomitant pathology	55	14
2. Laboratory tests of blood and urine	identification of inflammatory processes	55	14
3. Physical examination and palpation of the urethra and the walls of the vagina (on a gynecological chair)	assessment of the state of the external opening of the urethra, detection of the presence of any myofascial aspect in the disease	55	-
4. Uroflowmetry	assessment of the condition of the sphincters of the urethra and bladder	55	-
5. Transvaginal US/compression US	assessment of the size, shape, structure of the urethra and bladder neck/mapping of the urethral wall and surrounding tissues stiffness	24	6
6. Cystoscopy	examination the inside of the bladder in detail; identification and recording of abnormal findings	33 *	14 ^0^
7. CP OCT ^#^	visualization of the internal structure of the bladder neck and urethral wall, evaluation the condition of epithelium and connective tissue layers	33	14

* carried out to exclude the presence of interstitial cystitis; ^0^ patients with stones of the upper urinary tract but without pyelonephritis who have been assigned cystoscopy; ^#^ performed in conjunction with cystoscopy.

**Table 2:** Distribution of the CP OCT images by patient’s groups and parts of the urethra.

Group	Number of Patients	Number of CP OCT Images	Average Number of CP OCT Images Created from 1 Patient	Number of CP OCT Images of Each Location			
				Bladder Neck	Distal Urethra	Medium Urethra	Proximal Urethra
UPS	33	169	5.12	43	41	40	45
Norm	14	58	4.14	16	14	12	16
Total	47	227	4.63	59	55	52	61

**Table 3:** Concomitant pathology and the source of its occurrence in the group of patients with urethral pain syndrome (UPS) (*n* = 55).

Organ System with Pathology	n-Abs. (%)	Genesis of Pathology	n-Abs. (%)
1. Gynecological	39 (70.9)	Hormonal	37 (94.8)
		Inflammatory	30 (76.9)
		Surgical interventions on the pelvic organs	12 (30.7)
2. Respiratory	37 (67.2)	Upper (nose, nasal cavity, pharynx, larynx)	32 (86.4)
		Lower (trachea, bronchi, lungs)	5 (13.5)
		Psycho-emotional sphere	23 (41.8)
3. Neurological	35 (63.6)	Central nervous system	10 (18.2)
		Peripheral nervous system	42 (76.4)
		Psycho-emotional sphere	23 (41.8)
4. Urological	24 (43.6)	Inflammatory	10 (41.6)
		Non-inflammatory	17 (70.8)
5. Gastroenterological	18 (32.7)	Inflammatory diseases of the stomach, duodenum, biliary tract	38 (69.0)
		Bowel disease	21 (38.2)
6. Cardiovascular	9 (16.3)	Arterial hypertension	5 (55.5)
		Other	4 (44.5)
Total cases of pathology	162		

**Table 4:** State of the epithelium of the bladder neck and the proximal region of the urethra compared with the epithelium of the middle and distal regions at the ‘6 o′clock position’ in patients with UPS, depending on age.

Subgroup of Patients by Age, Years	Number of Patients	Number of CP OCT Images	Hyperplasia of the Bladder Neck + Proximal Urethra	Atrophy of the Bladder Neck + Proximal Urethra	Total Matches	% of Changes in the Bladder Neck + Proximal Urethra of the Total Number of Patients
≤49	18	72	4	4	8	44.4% (8/18)
50≥	15	60	7	7	14	93.3% (14/15)
Total (*n* = 33)	33	132	11	11	22	68.8% (22/33)
